# Oncogenic *RAS* instructs morphological transformation of human epithelia via differential tissue mechanics

**DOI:** 10.1126/sciadv.abg6467

**Published:** 2021-10-13

**Authors:** Agata Nyga, Jose J. Muñoz, Suze Dercksen, Giulia Fornabaio, Marina Uroz, Xavier Trepat, Buzz Baum, Helen K. Matthews, Vito Conte

**Affiliations:** 1 Institute for Bioengineering of Catalonia (IBEC), The Barcelona Institute of Science and Technology (BIST), Barcelona, Spain.; 2 MRC Laboratory of Molecular Biology, Cambridge, UK.; 3 Department of Mathematics, Polytechnic University of Catalonia (UPC), Barcelona, Spain.; 4 Centre Internacional de Mètodes Numèrics en Enginyeria (CIMNE), Barcelona, Spain.; 5 Institut de Matemàtiques de la UPC - BarcelonaTech (IMTECH), Barcelona, Spain.; 6 Department of Biomedical Engineering, Eindhoven University of Technology (TU/e), Eindhoven, Netherlands.; 7 Department of Physics, University of Barcelona (UB), Barcelona, Spain.; 8 Department of Biomedical Engineering and Biological Design Center, Boston University, Boston, MA, USA.; 9 Centro de Investigación Biomédica en Red en Bioingeniería, Biomateriales y Nanomedicina (CIBER-BBN), Barcelona, Spain.; 10 Department of Biomedicine, University of Barcelona (UB), Barcelona, Spain.; 11 Institució Catalana de Recerca i Estudis Avançats (ICREA), Barcelona, Spain.; 12 MRC Laboratory of Molecular Cell Biology, University College London (UCL), London, UK.; 13 Institute for Complex Molecular Systems (ICMS), Eindhoven University of Technology (TU/e), Eindhoven, Netherlands.

## Abstract

The loss of epithelial homeostasis and the disruption of normal tissue morphology are hallmarks of tumor development. Here, we ask how the uniform activation oncogene *RAS* affects the morphology and tissue mechanics in a normal epithelium. We found that inducible induction of *HRAS* in confined epithelial monolayers on soft substrates drives a morphological transformation of a 2D monolayer into a compact 3D cell aggregate. This transformation was initiated by the loss of monolayer integrity and formation of two distinct cell layers with differential cell-cell junctions, cell-substrate adhesion, and tensional states. Computational modeling revealed how adhesion and active peripheral tension induces inherent mechanical instability in the system, which drives the 2D-to-3D morphological transformation. Consistent with this, removal of epithelial tension through the inhibition of actomyosin contractility halted the process. These findings reveal the mechanisms by which oncogene activation within an epithelium can induce mechanical instability to drive morphological tissue transformation.

## Teaser

Uniform *RAS* activation in an epithelium induces a 2D-to-3D transformation through induction of mechanical instability.

## INTRODUCTION

Epithelia are layered tissues, which provide separation between the inside and external environment. To perform this barrier function, the organization of the epithelial tissues must be maintained as individual cells proliferate and die. Maintenance of epithelial homeostasis requires the dynamic regulation of cell proliferation, cell packing (*1*), and cell extrusion (*2*–*4*), the maintenance of a sheet-like morphology through the tight cell-cell association through intercellular junctions (*5*) and stable anchoring between cells and the extracellular matrix (ECM) (*6*).

Loss of homeostasis and the subsequent breakdown of tissue organization is a hallmark of epithelial-derived cancers, where oncogene activation results in the loss of carefully balanced control mechanisms. *RAS* oncogenes (*HRAS*, *NRAS*, and *KRAS*) are one of the most common mutated genes in human cancer with *RAS* activation occurring in around 30% of all cancers (*7*, *8*). *RAS* activation affects individual cell mechanics through alterations in actomyosin contractility (*9*–*12*). At the tissue scale, *RAS* disrupts epithelial homeostasis, resulting in loss of tissue polarity (*13*) and barrier function (*14*). Normal epithelia act as a barrier against single *RAS* cells or *RAS* cell clusters through their extrusion or delamination (*15*–*21*). The extrusion of *RAS* cells has been shown to be driven by actomyosin-dependent forces acting at the interface between normal and transformed cells (*18*, *20*) and can be affected by tissue-level mechanical forces (*17*). Larger clusters of *RAS* cells can separate from normal neighbors (*22*, *23*) to form cysts (*19*), outgrowths, or invaginations (*24*). Despite these morphological and mechanical roles for *RAS*, it is still not clear how cell and tissue mechanics mediate *RAS* signaling to morph epithelia independently of interactions between nontransformed and *RAS*-transformed cells.

Here, we investigated how the uniform activation of oncogenic *RAS* alters the morphology and mechanics of an epithelial monolayer and their cross-talk. In confined epithelial monolayers grown on soft physiological substrates, oncogenic *HRAS* expression was sufficient to transform the epithelial monolayer into a multilayered structure characterized by layer-specific differences in cell-cell contractility and cell-matrix adhesions. Furthermore, the increase in peripheral tension induced by oncogenic *HRAS* destabilized tissue mechanics to set the epithelia on a path to a three-dimensional (3D) morphological transformation.

## RESULTS

### Oncogenic *RAS* expression induces the active dewetting of confined MCF10A monolayers

**Fig. 1.**
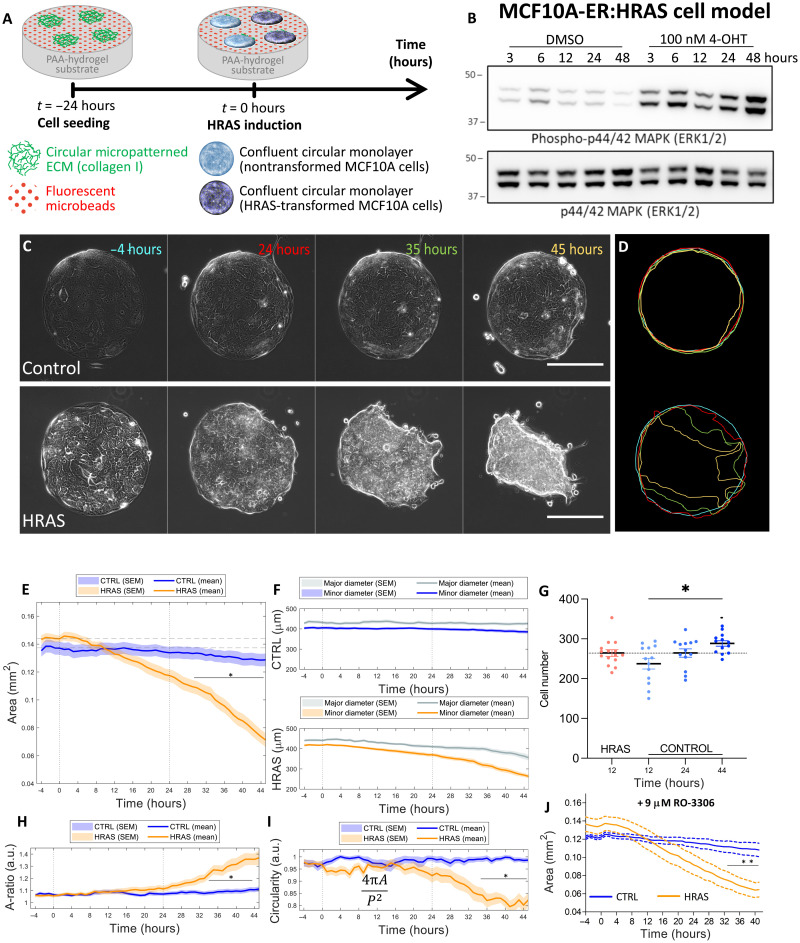
Morphological characterization of normal and *HRAS*-transformed MCF10A tissues. (**A**) Schematic of the experimental setup. (**B**) *HRAS* induction confirmed by increase in phosphorylation of mitogen-activated protein kinase (MAPK) (ERK1/2) shown in representative Western blots. (**C**) Phase contrast time-lapse of selected nontransformed (control) and *HRAS*-transformed MCF10A monolayers (imaging starts at *t* = −4 hours, and *HRAS* activation is induced at *t* = 0 hours; scale bars, 200 μm). (**D**) Contours of epithelia shown in (C) (time progresses with blue, red, green, and yellow colors). (**E**) Time evolution of the surface area of the epithelia’s footprint on the substate matrix (epithelial domain). (**F**) Time evolution of the major and minor diameters of the epithelia domain in the case of nontransformed and *HRAS*-transformed epithelia. (**G**) Cell number in *HRAS*-transformed epithelia at 12 hours after induction [start of area decline, (E)] compared to nontransformed epithelia at 12, 24, and 44 hours. Kruskal-Wallis test + Dunn’s multiple comparisons test. (**H**) Time evolution of the epithelial domain’s aspect ratio (i.e. major axis divided by minor axis of the epithelial domain). (**I**) Time evolution of the epithelial domain’s circularity. (**E** to **I**) Statistics over 15 nontransformed epithelia and 16 *HRAS*-transformed epithelia from at least four independent experiment repeats. Each epithelium was imaged for at least 50 hours. (**J**) Time evolution of the surface area of the nontransformed (*n* = 7) and *HRAS*-transformed (*n* = 8) epithelia domains during cell cycle arrest (9 μM RO-3306). (E, F, and H to J) Time evolution graphs represent means ± SEM of medians at each time point. Two-way analysis of variance (ANOVA) with Bonferroni posttest, *P < 0.05. a.u., arbitrary units.

To systematically study the effects of *RAS* activation on epithelial morphology and mechanics live (Fig. 1A), we conditionally activated oncogenic *HRAS* in nontransformed MCF10A human epithelial cells, MCF10A/ER:HRAS^V12^ (*25*). We modeled the physiological stiffness of basement membrane and underlying ECM by plating cells on soft polyacrylamide (PAA) gel substrates with a stiffness of 12 kPa (*26*, *27*). We coated substrates with rat tail collagen type I micropatterned in circular shapes of 400 μm in diameter (Fig. 1A). Epithelial cells were grown on these soft circular micropatterned substrates for 24 hours. At the time point *t* = 0 hours, the expression of *HRAS.V12* was induced in the epithelial monolayers through the addition of 4-hydroxytamoxifen (4-OHT) (*HRAS*-transformed epithelia). The vehicle control [dimethyl sulfoxide (DMSO)] was added to the remaining cellular monolayers as a control (nontransformed epithelia). Unexpectedly, we found that *HRAS* activation (Fig. 1B) was sufficient to transform the 2D epithelial monolayer into a 3D mass (Fig. 1, C and D, and fig. S1).

**Fig. 2.**
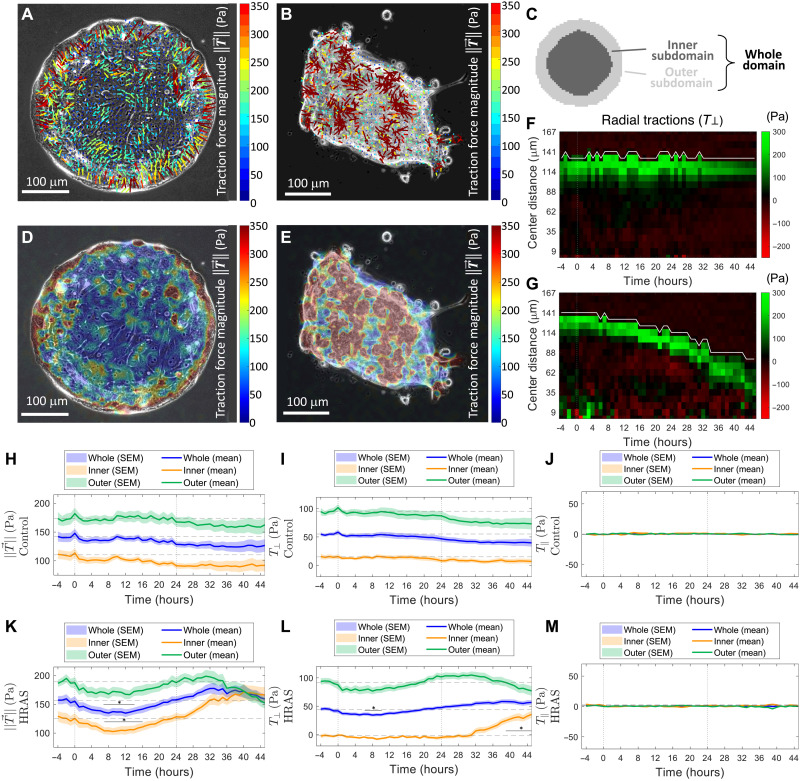
Mechanical characterization of normal and *HRAS*-transformed MCF10A tissues. Overlays of traction-force vectors (**A** and **B**) and traction-force magnitude maps (**D** and **E**) on phase contrast images of confined MCF10A epithelia. (A and D) Representative nontransformed epithelium at *t* = 19 hours. (B and E) Representative *HRAS*-transformed epithelium at *t* = 45 hours. (**C**) Schematic representing the whole epithelial domain along with its outer and inner subdomains. (**F** and **G**) Kymographs of the radial (perpendicular) component of the traction field *T*_⊥_: (F) for a representative nontransformed MCF10A epithelium and (G) for a representative *HRAS*-transformed MCF10A epithelium. White lines represent the average evolution of the edge of the island in time, the center of the island colocalizing with the bottom of the graph. A negative component *T*_⊥_ (red) means that the corresponding traction-force vector is oriented toward the exterior of the epithelial domain’s edge, whereas a positive component *T*_⊥_ (green) is indicative of traction-force orientation toward the interior of the epithelial domain’s edge. (**H** to **M**) Time evolution of magnitudes and components of the traction-force field in the whole epithelial domain (blue) and in the inner (orange) and outer (green) epithelial subdomains. (H and K) Time evolution of the average traction field ± magnitude for (H) nontransformed epithelia and (K) *HRAS*-transformed epithelia. Time evolution of the average traction force components: (**I** and **L**) perpendicular to the epithelial domain’s edge (*T*_⊥_) for (I) nontransformed and (L) *HRAS*-transformed epithelia and (**J** and **M**) tangential to the epithelial domain’s edge (T_∥_) for (J) nontransformed and (M) *HRAS*-transformed epithelia. (H to M) Statistics over 15 nontransformed epithelia and 16 *HRAS*-transformed epithelia from at least four independent experiment repeats. Each epithelium was imaged for at least 50 hours. Median over each epithelial domain at each time point of its evolution. Time evolution graphs represent means ± SEM of medians at each time point. Two-way ANOVA with Bonferroni posttest, *P < 0.05.

To define the path of this 2D-to-3D cellular transformation, we monitored the process via a set of objective morphological (Fig. 1) and mechanical measures (Fig. 2). Specifically, we quantified the area (Fig. 1E), aspect ratio (Fig. 1H), circularity (Fig. 1I), and traction forces (Fig. 2) for each cellular island.

Morphological analyses revealed differences between RAS and control epithelial monolayers beginning ~4 hours after *HRAS* induction. Whereas control monolayers remained flat and maintained a near-constant area throughout the time window of our analysis, from 4 hours after oncogenic *HRAS* induction, monolayer islands exhibited a slow decrease in area. This decrease in circular area continued in an approximately symmetric fashion up until 24 hours after *HRAS* induction, after which the symmetry of *HRAS*-transformed islands started to break (Fig. 1, E, F, H and I). At this point, *HRAS*-transformed monolayers underwent a loss of circularity and an increase in aspect ratio (Fig. 1, H and I). This was followed by a rapid transition as the 2D *HRAS-*transformed epithelium started to morph into a 3D cell mass (fig. S1). None of these changes were seen in control nontransformed monolayers. To study the impact of cell proliferation induced by *HRAS* on this behavior, we compared the number of cells in *HRAS*-transformed epithelia at 12 hours (when the cellular island is still a monolayer) with the number of cells in nontransformed epithelia over the span of the time window of our analysis. By 12 hours, the area of *RAS-*transformed monolayers had started decreasing substantially compared to pretransformation levels. By contrast, the increase in number of cells in nontransformed epithelia was not accompanied by a substantial decrease in surface area (Fig. 1, E and G). In addition and contrary to previous reports (*28*, *29*), inhibiting cell proliferation (cell cycle arrest via RO-3306 treatment; Fig. 1J) did not prevent 2D *RAS*-transformed monolayers morphing into a 3D cell mass (Fig. 1J). Together, these results indicated that cell proliferation did not drive the 2D-to-3D morphological transition undergone by *HRAS*-transformed monolayers.

Mechanical analyses carried out using traction force microscopy (*30*) showed that elevated traction forces were mainly localized at the periphery of epithelial islands. This was the case for both nontransformed and *HRAS*-transformed monolayers (Fig. 2, A, D, H, and K). In both cases, the elastic strain energy transferred by the epithelium to the underlying substrate followed a similar trend (fig. S2). The accumulation of traction force at the periphery of circular epithelial islands is in keeping with previous theoretical work (*31*, *32*) and experimental data from studies of human colon carcinoma cells (HCT-8) (*33*) and on confined circular monolayers of canine kidney cells (MDCK) (*34*, *35*).

Traction forces tended to be more ordered at the epithelium’s periphery (Fig. 2, A and B). To better understand this, we decomposed the traction field along the directions normal and tangential to the epithelial domain’s edge (fig. S3) and computed the net radial (*T*_⊥_) and net tangential (*T*_∥_) traction force components as a function of time in the whole epithelial domain and in the central (inner) and peripheral (outer) domains (Fig. 2C). These time trends (Fig. 2, I, J, L, and M) confirmed that the net physical interactions at the interface between epithelia and substrate developed along the direction perpendicular to the epithelium’s edge for both nontransformed and *HRAS*-transformed epithelia throughout the analysis.

To average out spatial-temporal fluctuations of the traction force field and better visualize reproducible force patterns, we averaged the net radial traction components *T*_⊥_ in space along lines concentric to the edge of the island. Upon displaying these space averages as a function of distance from the epithelial domain’s edge, we obtained kymographs of the net radial traction component *T*_⊥_ for both nontransformed (Fig. 2F) and *HRAS*-transformed MCF10A epithelia (Fig. 2G). Kymographs confirm that it is the intense net radial traction components that concentrate mainly at the periphery of both nontransformed and *HRAS*-transformed epithelia throughout the experiment. By analyzing the stress distribution within the epithelial domain through monolayer stress microscopy (Materials and Methods) (*30*), we further showed that nontransformed epithelia successfully establish and maintain epithelial homeostasis by means of long-range transmission of physical forces throughout the monolayer from opposite edges of the epithelium, which results in tension accumulating throughout the epithelial island (fig. S4).

The traction force analysis also revealed a characteristic two-phase oscillation in the traction field during the course of our analysis (Fig. 2K) that was specific to *HRAS*-transformed epithelia. Forces first decreased over a period of approximately 8 hours and then increased by 24 hours following *HRAS* induction (Fig. 2, K and L). Epithelial islands then underwent an abrupt 2D-to-3D morphological transformation (Fig. 1B and fig. S1), which was accompanied by rapid morphological (Fig. 1) and mechanical (Fig. 2) changes and is reminiscent of previously described cellular process of active dewetting (*36*), in which the tissue retraction is driven by a monotonical increase in tension and in cell-matrix traction forces oriented toward the center of the circular monolayer. These behaviors were not peculiar to *HRAS*, as the overexpression of *KRAS*^V12^ led to similar results. Upon *KRAS*^V12^ activation, confined circular monolayers of inducible MCF10A/ER:KRAS^V12^ cells followed a similar morphomechanical fate (fig. S5). The reproducible order of events observed following oncogenic *RAS* induction led us to hypothesize that oncogenic *RAS^V12^* primes epithelia for dewetting in the first 24 hours by inducing structural and mechanical changes within the monolayer while it is still morphologically flat.

### Oncogenic *HRAS* expression triggers the bilayering of confined MCF10A monolayers

**Fig. 3.**
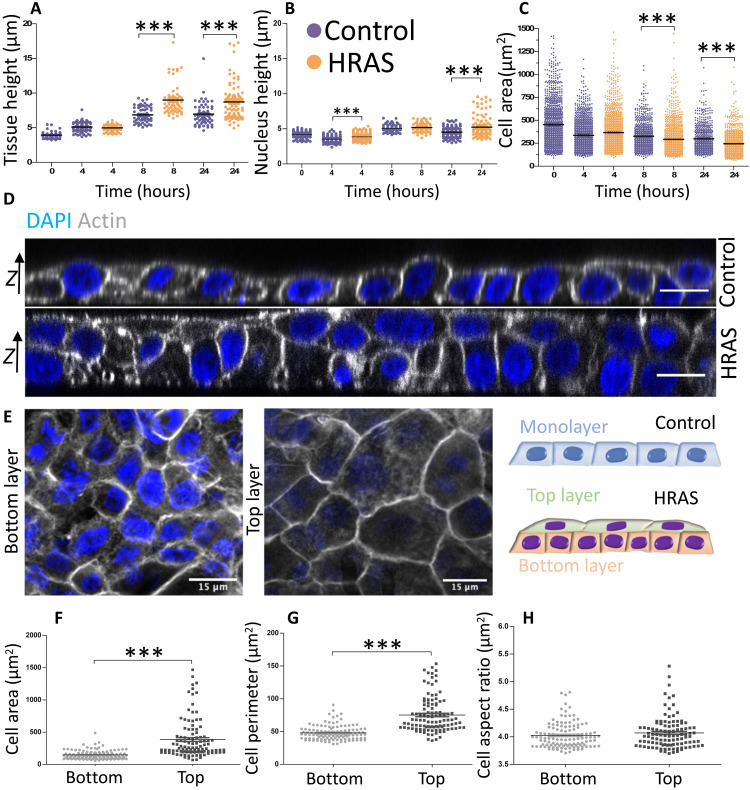
Oncogenic *HRAS*-expression induces the bilayering of MCF10A monolayers. (**A** to **C**) Measurements of epithelial monolayer features at selected time points for both nontransformed and *HRAS-*transformed epithelia: (A) tissue height, (B) nucleus height, and (C) cell area (in μm^2^). Kruskal-Wallis statistic test with Dunn’s multiple comparison test. (**D**) Representative confocal images of nontransformed monolayer (top) and *HRAS-*transformed bilayer (bottom) at *t* = 24 hours from oncogene induction. Focal planes are orthogonal to the substrate matrix (*z* axis). Actin is labeled in gray, and nuclei are labeled in blue. Scale bars, 15 μm. (**E**) Representative confocal image of nontransformed monolayer and *HRAS*-transformed bilayer. Focal plane crosses the tissue parallel to the substrate matrix. Actin is labeled in shades of gray, and nuclei are labeled in blue. Scale bars, 15 μm. (**F** to **H**) Quantification of cell features in the top and bottom layers of *HRAS*-transformed bilayers: (F) cell surface area, (G) cell surface perimeter, and (H) cell shape index. Means ± SEM of median values from at least three individual patterns. Mann-Whitney *U* test, ***P < 0.001.

To test this idea, we analyzed cellular organization in the *z* plane orthogonal to the underlying substrate at early stages following *RAS* induction. This revealed early changes in both nuclear and monolayer height (Fig. 3, A and B). Thus, within 24 hours of oncogene activation, *RAS-*transformed epithelia became an average of 25% thicker than nontransformed ones (Fig. 3A and fig. S6). This was accompanied by an increase in cell packing that was markedly higher in the confined *RAS-*transformed monolayers, leading to the observed decrease in the area of individual cells (Fig. 3C). The heights of nuclei in both nontransformed and *RAS-*transformed MCF10A monolayers were uniform up to 8 hours from oncogene activation (Fig. 3B). Then, between 8 and 24 hours, the heights of nuclei of *RAS-*transformed epithelia showed greater heterogeneity (*37*) as the *RAS*-expressing epithelium (but not the control) became multilayered (Fig. 3D). Notably, confocal microscopy of these epithelia revealed the segregation of confined *RAS-*transformed epithelia into two discrete cellular layers with very distinct organizations (Fig. 3, D and E). The cells forming the top layer of *RAS-*transformed epithelia were flatter and more spread out than those in the bottom layer (Fig. 3E), as measured by a 1.5-fold increase in the perimeter of cells (Fig. 3G) and a threefold increase in average cell area (Fig. 3F), without a concomitant change in aspect ratio (Fig. 3H). We next turned our focus on the cellular alterations that could cause the bilayering.

### Oncogenic *HRAS* expression induces layer-specific differences in cell-cell contractility and cell-matrix adhesions of the MCF10A bilayers

**Fig. 4.**
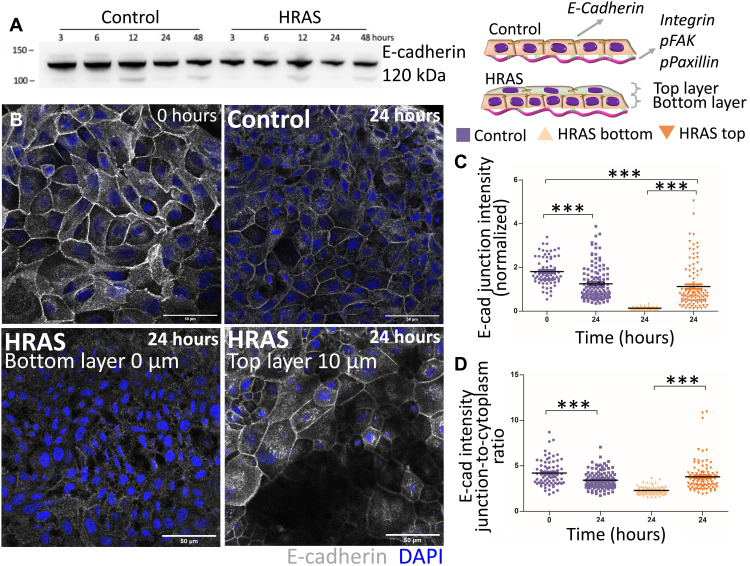
Oncogenic *HRAS* expression alters the expression of E-cadherin. (**A**) Western blot showing E-cadherin expression in nontransformed (control) and *HRAS*-transformed MCF10A cells over 48 hours from oncogene induction. (**B**) Representative confocal images showing the distribution of E-cadherin at *t* = 0 hours and within nontransformed and *HRAS*-transformed tissues at *t* = 24 hours. E-cadherin is labeled in gray, nuclei are labeled in blue. Scale bars, 50 μm. (**C** and **D**) Global E-cadherin fluorescence intensity in non-transformed (control) and *HRAS*-transformed epithelia: (C) junctional intensity (normalized) and (D) ratio of junctional to cytoplasmic intensity. Means ± SEM of median values from at least three individual patterns and Kruskal-Wallis statistic test with Dunn’s multiple comparison test, ***P < 0.001.

Stable RAS transformation has been shown to disrupt cadherins to promote cell invasion (*38*–*40*). Therefore, we hypothesized that the bilayering induced by oncogenic *RAS* expression might also be caused by disruption to cell-cell junctions. Nontransformed MCF10A cells constitutively expressed E-cadherin and, thus, exhibited uniform cell-cell junctions throughout the epithelium. Notably, E-cadherin expression did not change during *RAS* activation (Fig. 4A). Nevertheless, we observed differences in the localization of E-cadherin in the two layers of the developing bilayer (Fig. 4B). Cells in the bottom layer tended to have relatively low levels of E-cadherin at cell-cell junctions, while junctional E-cadherin levels remained similar to those before *RAS* activation in the top layer (Fig. 4C). In addition, the ratio of junctional to cytoplasmic E-cadherin was decreased in the bottom layer (Fig. 4D), suggestive of a redistribution of the protein away from junctions to cytoplasm. *RAS*-induced change in E-cadherin localization in the bottom layer was paralleled by a reduction of cell-matrix adhesion between the epithelial cells of this layer and the hydrogel substrate, as shown by a specific decrease in the expression of the collagen receptor integrin β1 (fig. S7, A to C). There was no difference in the expression of integrin β3 or β6 (fig. S7, D to F).

**Fig. 5.**
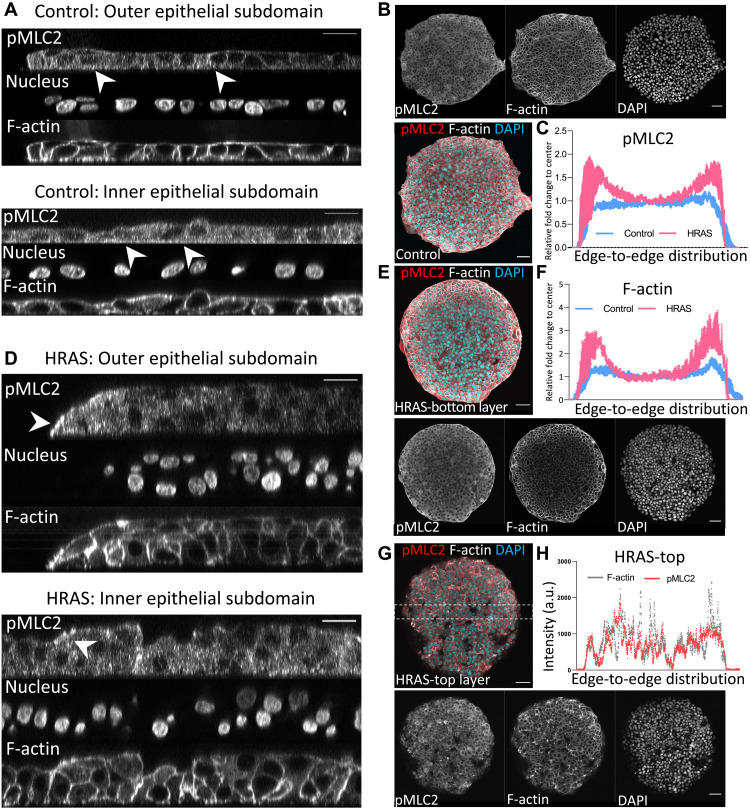
Oncogenic *HRAS* induction causes a tension differential in the MCF10A bilayers. Representative confocal images of nontransformed monolayer (**A**) and *HRAS-*transformed bilayer (**D**) at *t* = 24 hours from oncogene induction stained for pMLC2, F-actin, and DAPI (nuclei, shades of grays). Focal planes are orthogonal to the substrate matrix. Scale bar, 20 μm. (**B**, **E**, and **G**) Representative confocal images of entire nontransformed monolayer (B) and *HRAS*-transformed bilayer [(E) bottom layer and (G) top layer of the bilayer]. Focal plane crosses the tissue parallel to the substrate matrix. pMLC2, F-actin, and DAPI (nuclei) are labeled either in color code (red, gray, and blue, respectively) or shades of grays. Scale bars, 50 μm. (**C**) Relative fold change in averaged global intensity relative to center of the monolayer of pMLC2 fluorescence from edge to edge of the circular epithelial domain of nontransformed (blue) and *HRAS*-transformed (pink) monolayers (*n* = 3 and means ± S.D. in blue). (**F**) Relative fold change in averaged global intensity relative to center of the monolayer of F-actin fluorescence from edge to edge of the circular epithelial domain of nontransformed (blue) and transformed (pink) monolayers (*n* = 3 and mean ± S.D. in blue) and (**H**) individual averaged intensity profiles of pMLC2 (red) and F-actin (gray) of a representative section of *HRAS*-transformed top layer.

The structural alterations occurring in *RAS*-transformed epithelia at the level of cell-cell junctions (Fig. 4, A to E) and cell-matrix adhesions (Fig. 4, E to G) correlated with the initial decrease observed in traction forces transferred by the epithelium to the substrate matrix during the first ~8 hours of oncogene induction (Fig. 2K). However, traction forces recovered to pretransformation levels by 24 hours of *RAS* transformation (Fig. 2K), while adhesion to the substrate kept decreasing (Fig. 4, E to G). Since tension, measured by increase in ROCK (Rho-associated kinases), RhoA, and pMLC2 (phospho-myosin light chain 2), was previously associated with abnormal morphology of 3D malignant epithelial acini (*29*), we tested whether *RAS*-driven alterations to epithelial structure are reflected in changes in epithelial tension [driven by the contractile cellular actomyosin cortex (*41*, *42*)] by looking at the distribution of key cortex components F-actin and pMLC2 (*43*) in both nontransformed and *RAS-*transformed epithelia. While nontransformed epithelia show uniform distribution of F-actin and pMLC2 (Fig. 5, A to C), oncogenic *RAS* expression induced a gradient in the distribution of F-actin and pMLC2 throughout the bilayer within 24 hours of oncogene activation (Fig. 5, D to H). F-actin and pMLC2 fluorescent intensity were higher at the periphery of the bottom layer of *RAS-*transformed bilayers (Fig. 5, D to F). This fluorescent intensity was lower and more heterogeneously distributed throughout the top layer (Fig. 5, G and H), although spots of highly increased pMLC2 expression were visible at the periphery and in the middle of the top layer of *RAS-*transformed bilayers (Fig. 5G). Thus, these results show that while nontransformed monolayers retained a uniform homogenous state of tension (fig. S4) and organization (Figs. 1 and 2) as they grew under these conditions, oncogenic *RAS* expression led to the establishment of a tension gradient that destabilizes the epithelium within 24 hours, leading to a radial contraction (Fig. 1, E, F, H, and I) and the formation of multiple cell layers with very different properties (Fig. 2). To test whether this idea is physically plausible, we developed a simple computational model of circular cellular monolayer.

### Oncogenic *HRAS* expression makes confined monolayers mechanically instable

**Fig. 6.**
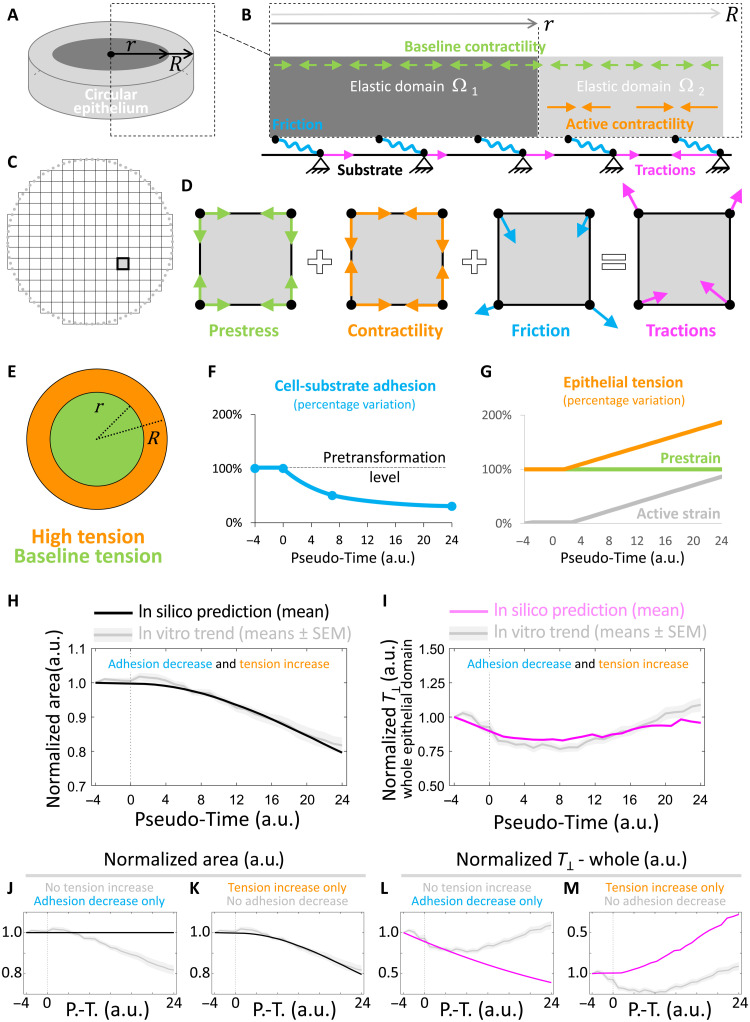
In silico model of *HRAS*-driven mechanical instability in MCF10A circular tissues. (**A** and **B**) Schematic of experimental features captured and emulated by the computational model (Materials and Methods). (**A**) Epithelium as a 2D continuum (grays) having bulk stiffness *E*, Poisson ratio ν, and finite thickness in plane-stress approximation. (**B**) Inter- and intracellular forces along with cell-matrix forces at the interface with the elastic substrate. Epithelium is uniformly subjected to a contractile prestrain constant in time (green arrows). Epithelial subdomain Ω_2_ (light gray) can further develop active strain. Active strain and prestrain develop contractile tensions equilibrating passive elastic forces within the epithelium and result in traction forces (magenta arrows) on the substrate matrix via solid viscoelastic friction (wavy blue elements). (**C**) Finite element discretization of the 2D epithelium. A representative finite element is highlighted. (**D**) Decomposition of forces acting at nodes of a representative finite element. (**E**) Epithelial contractile tension in epithelial subdomain Ω_2_ (orange) increases from baseline pretransformation levels of epithelial subdomain Ω_1_ (green) due to the combined effects of active strain and prestrain. (**F**) Adhesion with the substrate monotonically decreases from pretransformation levels in Ω_1_ and Ω_2_. (**G**) Evolution of epithelial tension (orange), active strain (gray), and prestrain (green). (**H** to **M**) Graphs comparing evolution of epithelial features in vivo (gray shaded lines) and in silico (i.e., model’s predictions). All trends have been normalized to time point *t* = −4 hours. (H, J, and K) Normalized epithelial surface area in silico (black line). (I, L, and M) Normalized traction force magnitude in silico (magenta line). (H and I) Case of both tension increase at the epithelium’s periphery and adhesion decrease throughout the epithelium. (J and L) Case of only adhesion decrease throughout the epithelium (no tension increase at the epithelium’s periphery). (K and M) Case of only tension increase at the epithelium’s periphery (no adhesion decrease throughout the epithelium).

The *RAS*-transformed circular epithelial bilayers were modeled in silico as a 2D continuum elastic disk having finite thickness in approximation of plain stress (Fig. 6A and Materials and Methods). The epithelial disk was mechanically coupled to the substrate matrix at discrete focal contact points (Fig. 6B). The effects of *RAS* induction were modeled on the basis of experimental data (Materials and Methods). The model emulated the dynamics of discrete focal contact points between cells of the monolayer and the substrate matrix by means of a viscoelastic solid friction law (*44*). Decreased adhesion between *RAS*-transformed bilayers and the underlying substrate matrix (Fig. 4, F to G) was modeled as a uniform value of solid friction that decreases in time (Fig. 6F and Materials and Methods). Increased cellular tension at the periphery of *RAS*-transformed bilayers, as observed in the experimental distributions of F-actin and pMLC2 (Fig. 5, C and F), was modeled as an increase in contractility (Fig. 6G) at the epithelial disk’s periphery (Fig. 6G). The increase in tension intensity was made proportional to the relative fluorescence intensities of pMLC2 (Fig. 5C and Materials and Methods). The finite element method was used to numerically resolve the bilayer’s deformations generated by active contractile cellular forces against friction of the in silico epithelial disk with the substrate (Fig. 6, C and D, and Materials and Methods). We then computed the profiles of the area of the epithelial disk and the traction force transmitted to the substrate (Fig. 6, H to M) as a function of cellular contractility (Fig. 6G) and/or cell-matrix adhesion (Fig. 6F).

Our simulations confirmed that an initial decrease in the adhesion between the epithelial disk and the underlying matrix can lead to a decrease in average traction force magnitude (Fig. 6I) and early contraction of the epithelium (Fig. 6H). However, despite cell-matrix adhesion further decreasing, our simulations showed that a progressive increase in epithelial tension led to the recovery of traction force magnitude to pretransformation levels (Fig. 6I) and further contraction of the epithelium (Fig. 6H). Thus, in combination, a decrease in cell-matrix adhesion followed by an increase in active epithelial tension, as observed experimentally (Figs. 4G and 5, C and F), is sufficient to emulate epithelial bilayer’s retraction and the oscillation of the traction force magnitude to the extents observed in vitro (Figs. 1E and 2, K and L). Our simulations show that the reduction in cell-matrix adhesion alone could only account for a monotonical reduction in traction-force magnitude (Fig. 6L), whereas the increase in cell-cell tension alone could only lead to a monotonical increase of traction-force magnitude (Fig. 6M) and decrease in epithelial area (Fig. 6L).

**Fig. 7.**
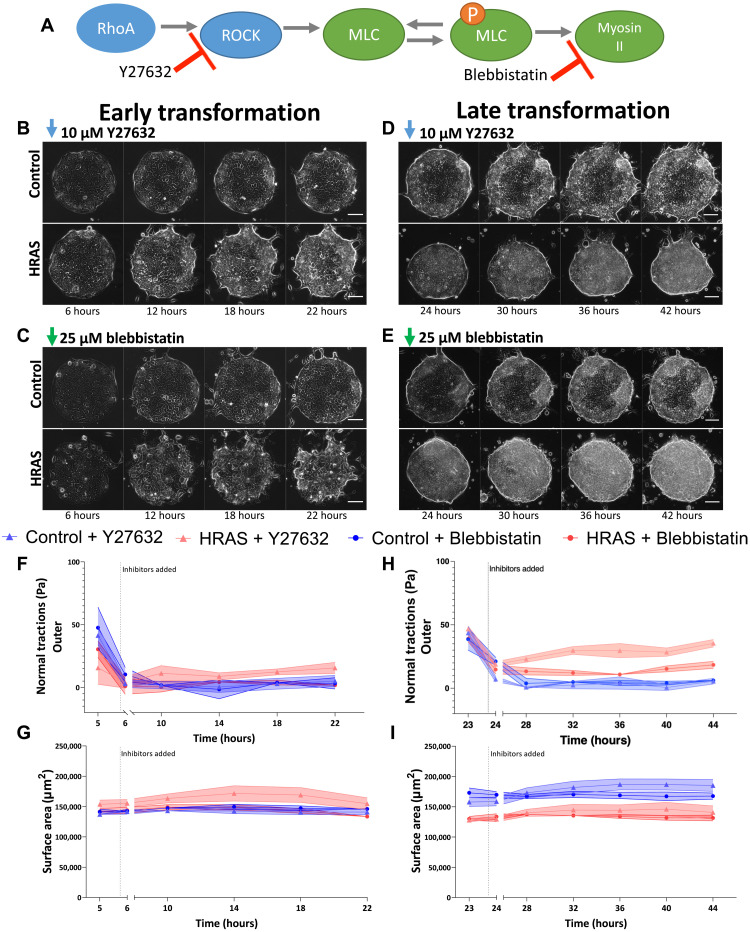
Targeting epithelial tension during *HRAS* transformation. (**A**) Schematic of the action of inhibitors. (**B** to **E**) Representative phase contrast images of confined nontransformed (Control) and *HRAS*-transformed MCF10A epithelia treated with ROCK inhibitor Y27632 (10 μΜ) (B and D) or myosin II inhibitor blebbistatin (25 μΜ) (C and E) during early transformation (6 hours) (B and C) or late transformation (24 hours) (D and E) Scale bars, 100 μm. (**F** and **H**) Time evolution of normal components of the traction-force field in the outer epithelial subdomains during early (F) and late (H) transformation in the presence of inhibitors. (**G** and **I**) Time evolution of the surface area of the epithelia’s footprint on the substate matrix during early (G) and late (I) transformation in the presence of inhibitors. Means ± SEM of medians at each time point (*n* = 3 to 5).

As a further experimental test of this idea, in experiments, we interfered with the build-up of epithelial tension at 6 hours (earlier stage; Fig. 7, B, C, F, and G) and at 24 hours (later stage; Fig. 7, D, E, H, and I) following *RAS* induction using pharmaceutical treatments that reduce epithelial tension. The inhibition of ROCK (Y27632; Fig. 7, A and B) or myosin II (blebbistatin; Fig. 7, A and C) at 6 hours prevented traction forces from recovering to pretransformation levels (Fig. 7F), preventing the contraction of epithelial monolayer (Fig. 7G). Similarly, the inhibition of ROCK (Y27632; Fig. 7, A and B) or myosin II (blebbistatin; Fig. 7, A and C) at 24 hours caused a drop in increasing traction forces (Fig. 7H) and prevented the morphological dewetting of the *RAS*-transformed bilayers at later time points (Fig. 7I). Hence, reduction of epithelial tension through ROCK or myosin II inhibition restored the mechanical stability of *RAS*-transformed epithelia within the time window of our analyses.

Together, our simulations and experiments suggested that *RAS* transformation induced a state of mechanical instability in epithelia during the first 24 hours of oncogene induction, where increased cell-cell tension is unbalanced by decreased cell-matrix adhesion. As a result, the homeostatic equilibrium of the *RAS*-transformed bilayers is lost, and this sets the bilayers on a path to a 3D morphological transformation.

## DISCUSSION

The *RAS*-genes superfamily functions as a crucial signaling hub within the cell, which controls several of its critical signaling cascades. This also reflects how profoundly the abnormal activity of *RAS* affects human development and disease (*7*, *8*, *45*). *RAS* mutations alter the morphology and mechanics of individual cells (*46*). In addition, as we show here, these morphomechanical changes are integrated at the tissue scale to induce profound changes in the organization of epithelia (Fig. 1). Thus, that uniform *RAS* induction induces changes in cellular morphologies and forces (Figs. 1 and 2), leading to the formation and contraction of a complex 3D cellular architecture via active dewetting (fig. S1).

This morphomechanical transformation is mediated by a series of changes in epithelial architecture and mechanics that are induced during the first 24 hours of uniform *HRAS-*oncogene induction (Fig. 3). These include (i) the establishment of two layers of cells that have very different patterns of an E-cadherin localization with high E-cadherin cell-cell junctions in the top layer and low E-cadherin cell-cell junctions in the bottom layer (Fig. 4); (ii) local reductions in cellular adhesion to the substrate (fig. S7), leading to decreased traction forces transferred by the affected tissues to the substrate (Fig. 2); and (iii) a redistribution of regulators of cellular tension (F-actin and pMLC2), which become concentrated at the periphery of the bilayer (Fig. 5). Our report of increased differential tension with reduction in adhesion during early oncogenic transformation contrasts the reports for fully malignant phenotypes, where increases in tension correlate with increases in focal adhesion formation (*47*). This highlights important differences between early and late malignant phenotypes. In the future, it will be fascinating to assess how the local changes in cell biology are induced by the uniform expression of oncogenic *RAS*. Our analysis shows how these local heterogeneities, when combined, give rise to the change in overall tissue organization and mechanics.

In a previous study, overexpression of E-cadherin in cancer cells set the epithelial monolayer for active dewetting (*36*). Here, we show that *HRAS* drives a decrease in cell-matrix adhesion and the redistribution of main regulators of epithelial tension, such as (i) F-actin and pMLC2, which became concentrated at the periphery of the bilayer (Fig. 5, C and F), and (ii) E-cadherin, which became differentially distributed between the top and bottom layers of the bilayer (Fig. 4). Our computational simulations show that this imbalance in cell-cell tension and cell-matrix adhesion brought about by oncogenic *RAS* expression is sufficient to place *RAS*-transformed epithelia into a state of mechanical instability (Fig. 6), which primes them for active dewetting (Fig. 1). Actively reducing tension through ROCK or myosin II inhibition further validated the role of the mechanical instability in this morphomechanical process.

Together, our findings demonstrate how activation of *RAS* oncogenes, even over short time periods, induces a mechanical imbalance in epithelial monolayers that results in the loss of homeostasis and a marked morphological transformation mimicking those seen in *RAS*-mutant human tumors. In pancreatic cancer, a mechanical imbalance following the oncogenic *KRAS* expression similar to the one studied here drives the pathological morphing of the pancreatic duct (*24*). Furthermore, increases in actomyosin tension in pancreatic ductal carcinoma also correlate with increased ECM remodeling and fibrosis (*48*), which are critical for oncogenic signaling to promote tumorigenesis (*49*). In squamous cell carcinoma, *HRAS* activation leads to layering via the loss of tissue polarity and organization (*50*). Here, we show that *RAS*-driven formation of two tissue layers with very different adhesive and contractile properties is a key event in the disruption of homeostatic equilibrium. By establishing a mechanical imbalance through *RAS*-transformed tissues, epithelial bilayering may also provide a favorable intermediate mechanism to abnormally morph epithelia without the need for an extended interface with nontransformed tissues (*19*, *22*–*24*, *50*). Overall, our findings establish a new physical mechanism of cellular collectives through which *RAS* can regulate the pathological morphology of epithelia autonomously of cell competition.

## MATERIALS AND METHODS

### Experimental design

To study mechanics in epithelial monolayers, we cultured epithelial cells on micropatterned PAA gels of defined stiffness (12 kPa). Detailed steps are described below.

#### MCF10A cell culture

Immortalized epithelial breast cell line MCF10A was transfected with inducible ^12^V-mutated form of the *HRAS* gene (a gift from Julian Downwards laboratory, University College London, London, UK) (*25*), referred to as MCF10A/ER.HRAS V12. They were maintained in complete medium composed of phenol-free Dulbecco’s modified Eagle’s medium (DMEM)–F12 medium (Thermo Fisher Scientific, #11039047) supplemented with 5% charcoal-stripped horse serum (Thermo Fisher Scientific, #16050122), penicillin (100 U/ml) and streptomycin (100 μg/ml; Thermo Fisher Scientific, #15070), epidermal growth factor (20 ng/ml; PeproTech, #AF100-15), hydrocortisone (0.5 mg/ml; Sigma-Aldrich, #H0888), cholera toxin (100 ng/ml; Sigma-Aldrich, #C8052), and insulin (10 μg/ml; Sigma-Aldrich, #I1882) (*51*) at 37°C in a humidified incubator with 5% CO_2_. Confluent cells were passaged every 2 or 3 days at 1:4 dilution.

#### Polyacrylamide (PAA) gel substrates

Glass-bottom six-well dishes (#0 thickness; IBL, #220.200.020) were treated with a Bind-Silane solution consisting of PlusOne Bind-Silane (VWR, #17-1330-01) and acetic acid (Panreac Quimica, #131008-1612) in absolute ethanol for 1 hour at room temperature (RT) in a fume hood. Wells were washed three time with ethanol and dried. PAA gels with a Young’s modulus of 12 kPa were prepared by mixing 18.8% of 40% (w/v) acrylamide (Bio-Rad, #1610140), 8% of 2% (w/v) bis-acrylamide (Bio-Rad, #1610142), 0.5% of 10% ammonium persulfate (Bio-Rad, #161-0700), 0.05% of *N*,*N*,*N*,*N*′-tetramethylethylenediamine (Sigma-Aldrich, #T9281), and 0.7% of FluoSpheres carboxylate modified microspheres (0.2 μm, dark red; Thermo Fisher Scientific, #F8807) in Hepes solution (Thermo Fisher Scientific, #15630056). Twenty-two microliters of solution was placed on the treated glass well and covered with an 18-mm-diameter coverslip. After 1-hour polymerization at RT, phosphate-buffered saline (PBS) was added to the wells and coverslips were carefully removed. Gels were washed with PBS.

#### Polydimethylsiloxane (PDMS) membranes

SU8-50 master containing circular patterns with diameter of 400 μm and height of 50 μm was prepared using conventional photolithography. Polydimethylsiloxane (PDMS) was spin-coated on the masters to a thickness lower than the height of the SU8 features (15 μm) and cured overnight at 85°C. A thick border of PDMS was left at the edges of the membranes for the handling purpose. PDMS membranes were peeled off and kept at 4°C until use.

#### Collagen patterning

To pattern collagen on top of PAA gels, gels were functionalized with a solution of Sulfo-SANPAH [1 mg/ml; sulfosuccinimidyl 6-(4′-azido-2′-nitrophenylamino) hexanoate; Thermo Fisher Scientific, #22589] for 5 min under ultraviolet lamp (XX-15, UVP) under 365-nm wavelength. After washing off remaining Sulfo-SANPAH with sterile PBS, gels were left to air-dry for 20 min inside a cell culture hood. PDMS membranes (nine patterns per membrane), passivated in 2% Pluronic F-127 (Sigma-Aldrich, #P2443) in ddH_2_O for at least 24 hours, were washed in PBS and air-dried inside a cell culture hood. PDMS membranes were placed on top of the PAA gels, and 50 μl of rat tail collagen type I solution (0.1 mg/ml; First Link, #112296) was placed on top of the patterns. PAA gels were incubated overnight at 4°C.

#### Monolayer patterning

PDMS membranes were removed from the top of the PAA gels by first adding sterile PBS. Gels were washed with PBS and incubated with 200 μl of PLL-g-PEG solution [0.1 mg/ml; PLL(20)-g[3.5]-PEG(2), SUSOS AG] for 30 min at 37°C. In the meantime, MCF10A cells were trypsinized and counted. Following incubation, gels were washed once with PBS and air-dried for 5 min. MCF10A cell suspension (50 μl) containing 50,000 cells was placed on top of the gel. Cells were incubated for 1 hour for attachment; the non-attached cells were washed three times with PBS, and cells were incubated in DMEM-F12 media for 24 hours in 5% CO_2_ at 37°C.

#### Drug treatment

After 24 hours of incubation, supernatant was aspirated and fresh DMEM-F12 medium containing 4-OHT (100 nM; Sigma-Aldrich, #H7904) was added to conditionally express *HRAS*, while equivalent amount of vehicle control, DMSO (1:1000, control), was added to control wells. For proliferation inhibition a cyclin-dependent kinase 1 inhibitor RO-3306 (Sigma-Aldrich, #SML0569) was used at 9 μM at the same time as 4-OHT/DMSO treatment and throughout the imaging. To inhibit epithelial tension, 10 μM ROCK inhibitor Y27632 (Sigma-Aldrich, #Y0503) or 25 μM myosin II inhibitor (**±**)-blebbistatin (Sigma-Aldrich, #203390) was added to media after 6 or 24 hours of *HRAS* induction (with 4-OHT still present throughout the experiments).

#### Western blot

To confirm *HRAS* induction with 4-OHT, total cell protein lysates were obtained by lysing cells exposed to 100 nM 4-OHT with radio-immunoprecipitation assay (RIPA) buffer (Thermo Fisher Scientific, #89900) containing phosphatase inhibitor cocktail 1 and 2 (Sigma-Aldrich, P5726 and P2850) and protease inhibitor cocktail (Sigma-Aldrich, #11836170001). Protein content was quantified with BCA Protein Assay kit according to the manufacturer’s instructions (Thermo Fisher Scientific, #23227), and 20 μg of protein was mixed with 2× Laemmli buffer (Sigma-Aldrich, #S3401) and boiled for 5 min at 95°C. Protein were separated using MOPS buffer (Thermo Fisher Scientific, #NP0001) in NuPAGE 4 to 12% bis-tris protein gels (Thermo Fisher Scientific, #NP0323BOX) at 150 V for 70 min at RT. Proteins were transferred to a nitrocellulose membrane at 100 V for 60 min at 4°C. Membranes were blocked for 30 min in 5% milk in tris-buffered saline with Tween 20 (TBST), followed by overnight incubation with primary antibodies diluted in 2.5% milk in TBST. Primary antibodies included rabbit phospho p44/42 mitogen-activated protein kinase (MAPK) (ERK1/2) (1:2000; Cell Signaling Technology, #4370S), rabbit p44/42 MAPK (Erk1/2) (1:2000; Cell Signaling Technology, #4695S), and mouse E-cadherin (1:1000; BD Biosciences, #610181). Primary antibodies were washed three times with TBST, followed by 1-hour incubation with secondary antibodies diluted in 2.5% milk in TBST [1:5000; goat anti-rabbit horseradish peroxidase (HRP) or goat anti-mouse HRP; DAKO, #P0448 and #P0447]. Membranes were washed three times with TBST and exposed to HRP substrate (Immobilon Crescendo, Millipore, #WBLUR0100) for chemiluminescence detection using ChemiDoc MP Imaging System (Bio-Rad).

#### Time-lapse microscopy

Multidimensional acquisitions were performed on an automated inverted microscope (Nikon Ti2 Eclipse, Nikon) using 20× objective (Nikon CFI Plan Apo 20X/0.75 Ph DM). Microscope was equipped with thermal, CO_2_, and humidity control and controlled using NIS software and perfect focus system. Images were obtained every 15 min over 50 hours. Up to 15 independent patterns were imaged in parallel using a motorized *XY* stage. Patterns where cells formed full monolayers at the start of experiments were selected for imaging.

#### Traction force microscopy

Gel displacements between any experimental time point and a reference image obtained after cell trypsinization were computed by using a custom-made particle imaging velocimetry code developed in the laboratory of X. Trepat by using 32-pixel resolution and overlap of 0.5. Traction forces were computed from hydrogel displacements through a custom-made code, which is based on a Fourier transform algorithm for elastic hydrogel substrates having finite thickness (*52*). Patterns for analysis were selected on the basis of quality of bead images, remained in focus throughout the imaging. To define the outer and inner domain, the binary mask of epithelia (identified by filling in their contour) was divided into concentric rings that are one single pixel in thickness. Rings are then labeled through integers 1 to *n* moving from the edge of the pattern toward its center. The external and internal subdomains are identified by grouping adjacent rings together; the first *m* rings from the edge (labels 1 to *m*) are merged to form the external subdomain, whereas the remaining *n-m* rings are merged to form the inner subdomain (the core of the disk). The label *m* is determined in such a way that both domains contain the same amount of pixels/data for unbiased statistical analyses over the two subdomains. The algorithm was implemented in MATLAB (license number 284992) for automatic execution on the individual mask of epithelia at each time point.

#### Monolayer stress microscopy

Intercellular and intracellular stresses in nontransformed MCF10A monolayers were computed via monolayer stress microscopy (*30*) via a custom software implemented via the custom finite element method platform EMBRYO developed in the laboratory of J.J. Muñoz. Briefly, the forces exerted by the elastic hydrogel substrate on the MCF10A epithelium (as a reaction to the traction force field transferred by the epithelium to the substrate) are equilibrated by the tensorial stress state within the epithelium. A necessary condition for the application of this technique is that epithelia maintain their monolayer architecture, a hypothesis only valid for nontransformed MCF10A monolayers in this study.

#### Immunofluorescence

Cells were fixed with 4% paraformaldehyde (Santa Cruz Biotechnology, #sc-281692) for 10 min and washed with PBS. Samples were incubated with block buffer containing 1% bovine serum albumin (Sigma-Aldrich, #A7906) and 0.3% Triton X-100 (Sigma-Aldrich, #T8787) in PBS at RT for 1 hour. Primary antibodies (mouse E-cadherin, 1:1500; BD Biosciences, #610181; mouse integrin β1, 1:250; Abcam, ab30394; rabbit pMLC2, 1:50; Cell Signaling Technology, #3671S; mouse integrin β3, 1:200; Merck, #MAB2023Z; mouse anti-CD61 (integrin β3), 1:200; eBioscience, #16-0611-82; mouse integrin αVβ6, 1:200; Merck, #MAB2077Z) were diluted in block buffer and incubated on top of samples overnight at 4°C. Subsequently, samples were incubated with secondary antibodies (fluorescein isothiocyanate (FITC) anti-mouse, 1:1000; Jackson ImmunoResearch, #715-545-150; Alexa Fluor 564 anti-rabbit, 1:500; Thermo Fisher Scientific, #A11035) for 2 hours at RT. F-actin was stained by incubating for 30 min with phalloidin-iFluor 594 CytoPainter (1:2000; Abcam, #ab176757) or Phalloidin-Atto 647N (1:1000; Sigma-Aldrich, #65906) at RT. In between steps, samples were washed with wash buffer [0.05% Tween 20 (Sigma-Aldrich, #P9416) in PBS]. Samples were covered with Fluoroshield mounting medium containing 4′,6-diamidino-2-phenylindole (DAPI) (Sigma-Aldrich, #F6057) and stored at 4°C until imaging.

#### Microscopy

Fluorescent images of the patterns were acquired with an inverted microscope (Nikon Ti2 Eclipse, Nikon) with an objective 20×/0.75 (Nikon CFI Plan Apo 20X/0.75 Ph DM). Confocal images were taken using inverted confocal microscope Axio Observer 7 (Spectral Detection Zeiss LSM 800) using 40×/1.3 Oil DIC M27 or 63×/1.4 Oil DIC M27 objectives with ZEN 2.3 imaging software. For integrin imaging, Zeiss Axiovert 200M microscope was used with 20× and 40× objective. For pMLC2 imaging, Zeiss LSM 780 microscope was used with 20×/0.8 M27 Plan-Apochromat and 40×/1.20 W Korr M27 C-Apochromat objectives using ZEN 2.1 SP3 software.

#### Nanoindentation

Monolayer stiffness (Young’s modulus) was measured by means of the Piuma Nanoindenter (from Optics 11) fitted with a cantilever having stiffness of 0.05 N/m and spherical tip with a radius of 10 μm. Four measurements were taken from three different samples. The Young’s modulus of epithelia before oncogene induction resulted in 1.363 ± 0.504 kPa (mean ± SD).

#### Image analysis

To detect the physical properties of the epithelial monolayer from fluorescent images, a pipeline was created in CellProfiler (*53*) and followed by postprocessing of the images and data in custom-made automatic workflow in MATLAB (license number 284992). Images of nuclei and F-actin were used to detect both the individual nuclei and cell borders within monolayers. The intensity of the images was rescaled to the full range of the intensity histograms (minimum, 0; maximum, 1), and uneven illumination was corrected by subtracting a spline illumination function. Nuclei were segmented using the adaptive Otsu three-class thresholding with the middle intensity class assigned as the background. To improve detection, we optimized minimum diameter (16-pixel unit) and threshold correction factor (0.8). Clumped objects were distinguished by intensity and cell outlines by propagation method. The precision of nuclei detection was assessed by comparing the outcomes of the pipeline with manual nuclei detection in ImageJ. Features extracted from the image processing included cell ID, nuclei center coordinates, areas, and perimeters. Using custom-made workflow in MATLAB (license number 284992), we calculated cell and nuclei shape indices and removed outliers (based on surface areas, shape index, and nucleus areas).

#### E-cadherin analysis

To measure E-cadherin intensity, a line was drawn using Fiji ImageJ (version 1.53c) (*54*) between two nuclei. The intensity range was then normalized by subtracting the 1st percentile and dividing by 99th percentile. The junctional intensity was extracted, and the ratio between junctional and cytoplasmic intensity was calculated by dividing the junctional value by average of the cytoplasmic value.

#### Integrin analysis

To measure integrin intensity, a global intensity value of the total image was obtained with ImageJ.

#### pMLC2 analysis

Fluorescence intensity was determined using Fiji. Rectangular shape (531.37 μm by 290 μm) was placed from one end of the image to other encompassing the middle part of the pattern, and the average intensity was measured. The background intensity was removed by calculating intensity in area outside the pattern for each image. Data from three independent patterns were presented as means ± SE.

#### Data analysis

To perform statistical analysis, GraphPad Prism (version 9.0.0) was used. Data distribution was assessed using D’Agostino and Pearson omnibus normality test. Data from all conditions had to pass the normality test to be included in parametric testing: for nonparametric data, (i) two groups, Mann-Whitney *U* test; (ii) more than two groups, Kruskal-Wallis statistic test used with Dunn’s multiple comparison test; (iii) two groups over long time course, two-way analysis of variance (ANOVA) with Bonferroni post-test; and (iv) one condition over long time course, Friedman test with Dunn’s multiple comparison test. *P* < 0.5 indicated a statistical significance (*P < 0.05, **P < 0.01, and ***P < 0.001).

#### Computational model

The evolution of the net radial traction components is modeled by resorting to a 2D finite element model of the flat tissue. The flat tissue is represented by a circular domain Ω with radius *R* with two distinct subdomains: subdomain Ω_1_ subjected to a constant baseline contractile force $\varepsilon_{0}^{c}$ (prestrain) and subdomain Ω_2_ subjected to an additional active contractile strain ε*^c^* for a total contractile strain of $\varepsilon =\varepsilon_{0}^{c}+\varepsilon^{c}$ (Fig. 6B). The radius of the central subdomain Ω_1_ decreases in time from *R* (pretransformation levels) to r=R3 to emulate the shape of the pMLC2 trend (Fig. 5C). The underlaying substrate is modeled as a set of nodal locations fixed in time. The elastic domains Ω_1_ and Ω_2_ develop local tension because of these prescribed strains. Moreover, each domain is subjected to a specific degree of a solid viscoelastic adhesion with the underlying substrate (*44*), which is specifically modeled via a Kelvin-Voight model. Weakening of cell-matrix adhesion is simulated by applying a reduction factor α to the cell-matrix adhesion constant κ. The subdomains Ω_1_ and Ω_2_ are assumed to have linear elastic behavior, and tissue motion is determined in the approximation of quasi-static equilibrium; there, the active contractility ε*^c^* and the baseline prestrain $\varepsilon_{0}^{c}$ equilibrate the passive elastic forces within the tissue and the adhesion forces with the substrate. Model’s deformations in each subdomain Ω_1_ and Ω_2_ were determined via Cauchy’s equation

∇·σ+f=0 (1)

where σ is the elastic stress tensor given by **σ** = λ trace(**ε***^e^*) + 2μ**ε***^e^*, **ε***^e^* is the elastic strain, with $\varepsilon^{e}=\varepsilon -\varepsilon_{0}^{c}I$ in Ω_1_ and $\varepsilon^{e}=\varepsilon -(\varepsilon_{0}^{c}+\varepsilon^{c})I$ in Ω_2_, λ = *E*ν/((1 + ν)(1 − 2ν)) and μ = *E*/(2(1 + ν)) are the Lamé elasticity constants, E is the Young modulus and ν the Poisson ratio of both subdomains Ω_1_ and Ω_2_, ***f*** = α*k****u*** is the adhesion force between the tissue and the substrate, ***u*** is the tissue displacement and *u_i_* is its component, ε*_ij_****=*** 0.5(*∂_i_u_j_* + *∂_j_u_i_*) is the total (observable) strain, and α is the weakening factor of the tissue adhesion forces with the substrate.

We use the finite element method to turn Eq. 1 into a linear system of equations

$$(K+K_{s})u=f$$ (2)

where **K** and **K**_***s***_ are the stiffness matrices corresponding to the elastic and adhesive contributions, respectively.

The force vector **f** in Eq. 2 represents the resulting contractile force due to active contractile strain ε*^c^* and/or baseline prestrain $\varepsilon_{0}^{c}$. The time evolution of α, ε*^c^*, and $\varepsilon_{0}^{c}$ are given in table S1. Vector **f** includes the assembling of vectoral contributions **f**_**1**_ and **f**_**2**_, respectively, from the subdomains Ω_1_ and Ω_2_, with

$$f_{1}=\varepsilon_{0}^{c}\int_{\Omega_{1}}B^{T}D{\left\{1\;1\;0\right\}}^{T}\;d\Omega \;\text{and}\;f_{2}=(\varepsilon_{0}^{c}+\varepsilon^{c})\int_{\Omega_{2}}B^{T}D{\left\{1\;1\;0\right\}}^{T}\;d\Omega$$

where **B** is the deformation matrix such that **ε** = **Bu** and **D** is the elasticity matrix that depends on *E* and ν.

Last, the discretization of the finite element mesh used 7860 square elements (100 elements along the diameter). We then used experimental data to set (i) the bulk stiffness of the epithelium as having a value of 1 kPa before *HRAS* activation; this is based on nanoindentation measurements of MCF10A epithelia having an in vitro stiffness of 1.36 ±0.5 kPa (see Materials and Methods). Furthermore, for the sake of simplicity, we also set (ii) the Poisson’s Ratio of the tissue elasticity to ν = 0.46 (quasi-incompressibility), (iii) the drop in adhesion with the substrate to a monotonic decrease (Fig. 6, B and F) emulating the average drop in the expression of the collagen receptor integrin β1 within 24 hours from *RAS* activation (fig. S7, B and C), and (iv) a linear increase in local contractile strain $\varepsilon =\varepsilon_{0}^{c}+\varepsilon^{c}$ (Fig. 6, B and G) to a value approximately double that of the homeostatic baseline; this is based on combined pMLC2 fluorescence intensity from bottom and top layers of the *RAS-*transformed bilayer (Fig. 5, F and H) that approximately amounts to double that of nontransformed monolayers (Fig. 5C). All model’s parameters are summarized in table S1.

## Notes

View/request a protocol for this paper from *Bio-protocol*.
