# Supplementary material for: Oncogenic RAS instructs morphological transformation of human epithelia via differential tissue mechanics
Source: Sci Adv. 2021 Oct 13;7(42):eabg6467. doi: 10.1126/sciadv.abg6467 (PMC8514103; doi:10.1126/sciadv.abg6467)
Supplement: Supplementary file 1 — Figs. S1 to S7 Table S1 Legends for movies S1 to S4 [file sciadv.abg6467_sm.pdf]

Science Advances
AAAS

# Supplementary Materials for

## Oncogenic *RAS* instructs morphological transformation of human epithelia via differential tissue mechanics

Agata Nyga, Jose J. Muñoz, Suze Dercksen, Giulia Fornabaio, Marina Uroz, Xavier Trepat, Buzz Baum, Helen K. Matthews, Vito Conte*

*Corresponding author. Email: v.conte@tue.nl

Published 13 October 2021, *Sci. Adv.* **7**, eabg6467 (2021)
DOI: 10.1126/sciadv.abg6467

**The PDF file includes:**

Figs. S1 to S7
Table S1
Legends for movies S1 to S4

**Other Supplementary Material for this manuscript includes the following:**

Movies S1 to S4

**Fig. S1.**

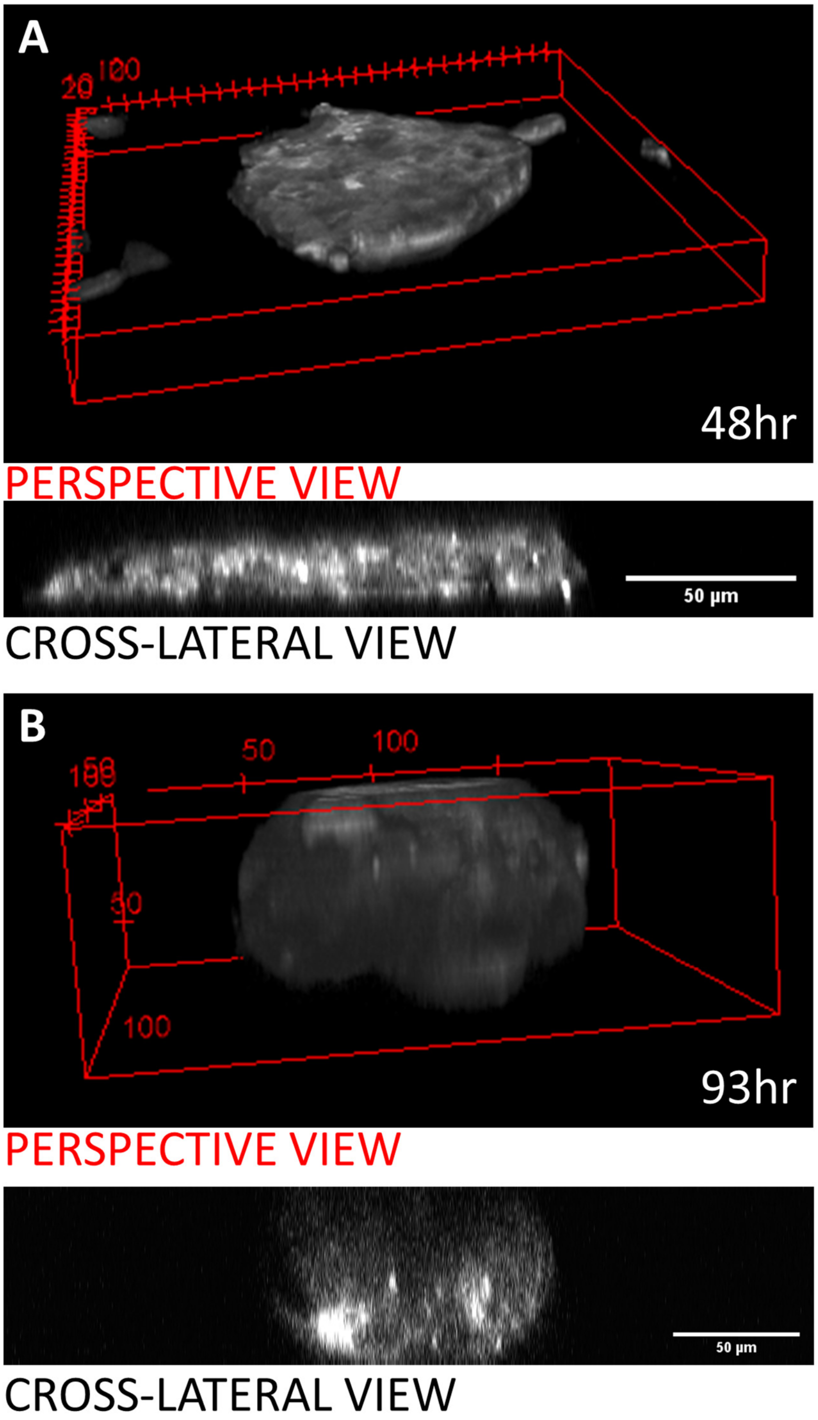


**The HRAS cell mass.**
**A-B)** Confocal microscopy reconstruction of HRAS-transformed 3D cell mass at t=48 (A) and t=93 hours (B) both in perspective and cross-lateral view.

**Fig. S2.**

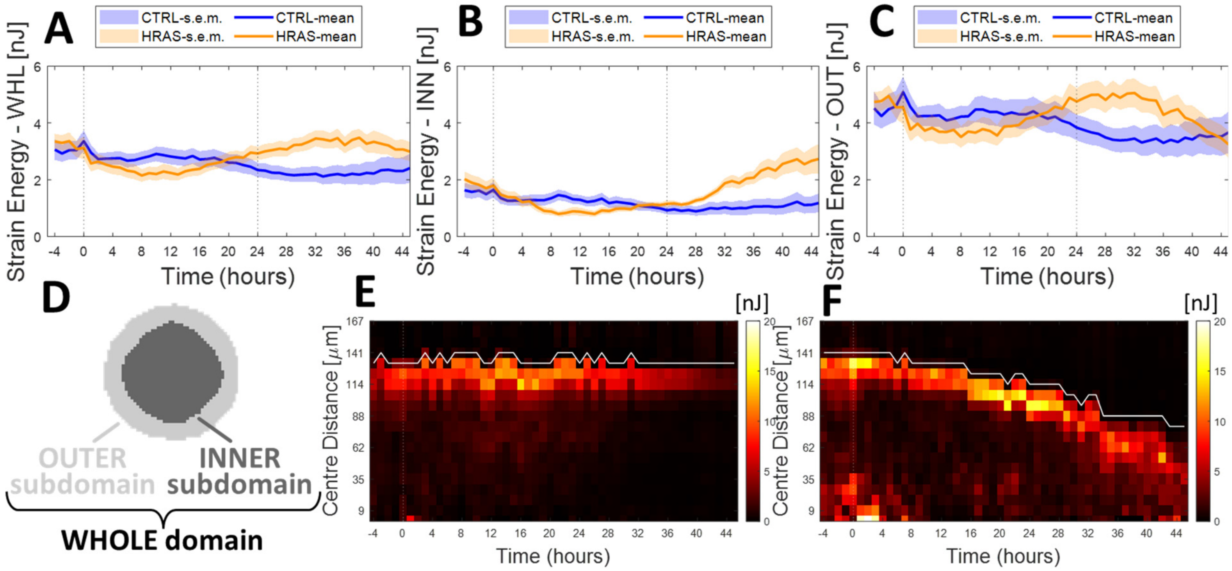


**Strain Energy. A-C)** Time-evolution (Mean±S.E.M.) of the elastic strain energy intensity actively transferred by the epithelium to the elastic gel substrate (Mean±S.E.M). Elastic strain energy was defined as one-half the scalar product between cellular traction and the gel displacement that it generates. Statistics over 15 non-transformed epithelia and 16 HRAS-transformed epithelia from at least 4 independent experiment repeats. Strain energy trends in: **A)** the whole island's domain; **B)** inner island's subdomain; and, **C)** the outer island's subdomain. **D)** Schematic representing the whole epithelial domain along with its outer and inner subdomains. **E-F)** Kymographs of the strain energies: **E)** for a representative control MCF10A circular epithelium; and, **F)** for a representative *HRAS*-transformed MCF10A circular epithelium. White lines represent the average evolution of the edge of the island in time, the center of the island co-localizing with the bottom of the graph.

**Fig. S3.**

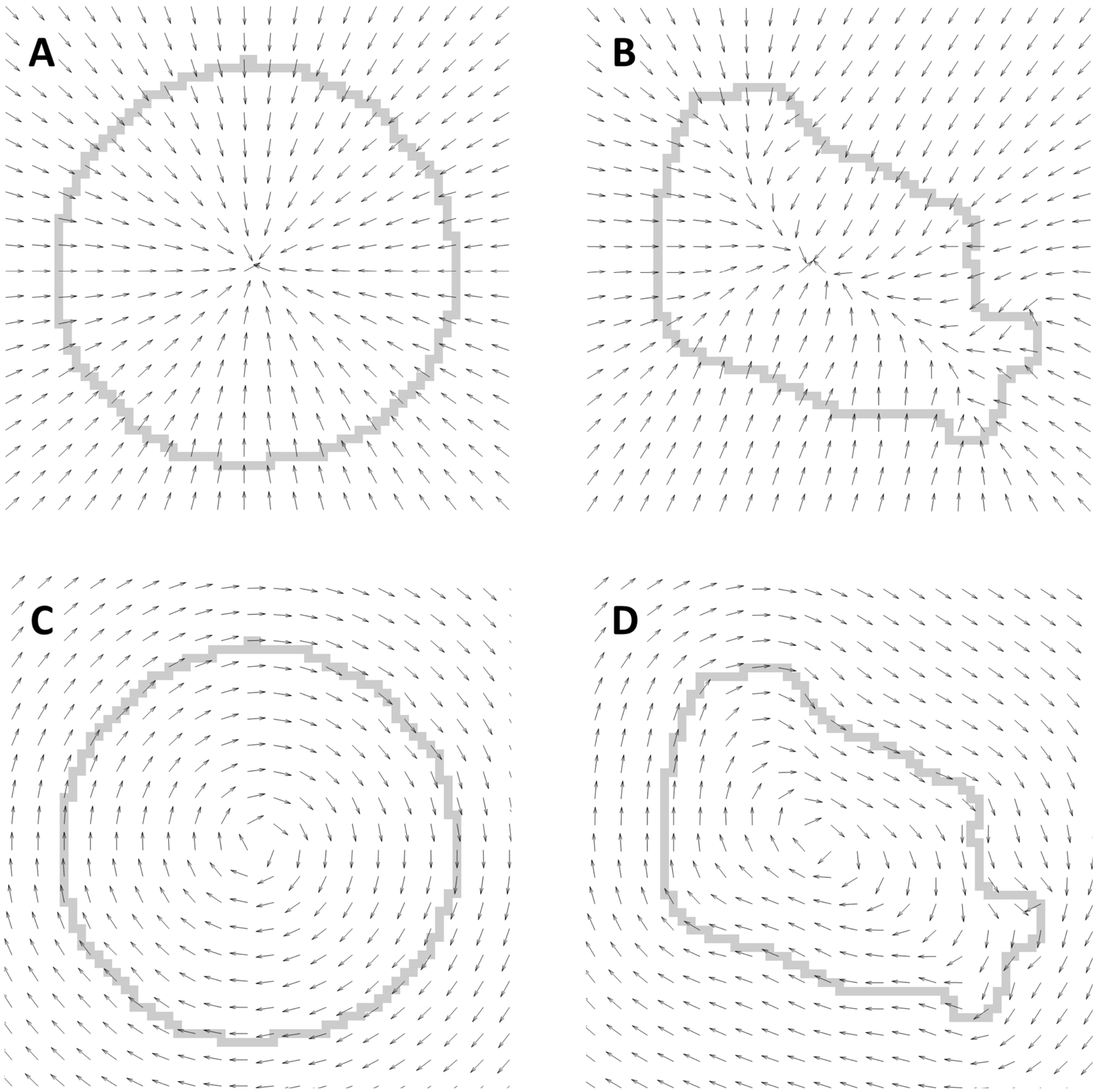


**Vector fields of normal and tangent directions. A-D)** Representative diagrams showing the mutually orthogonal fields of unit directions (black arrows) that are: **A-B)** perpendicular to the epithelial domain's edge (grey) in an earlier (A) and later (B) stage of epithelial evolution; and, **C-D)** tangential to the epithelial domain's edge (grey) in an earlier (C) and later (D) stage of epithelial evolution.

**Fig. S4.**

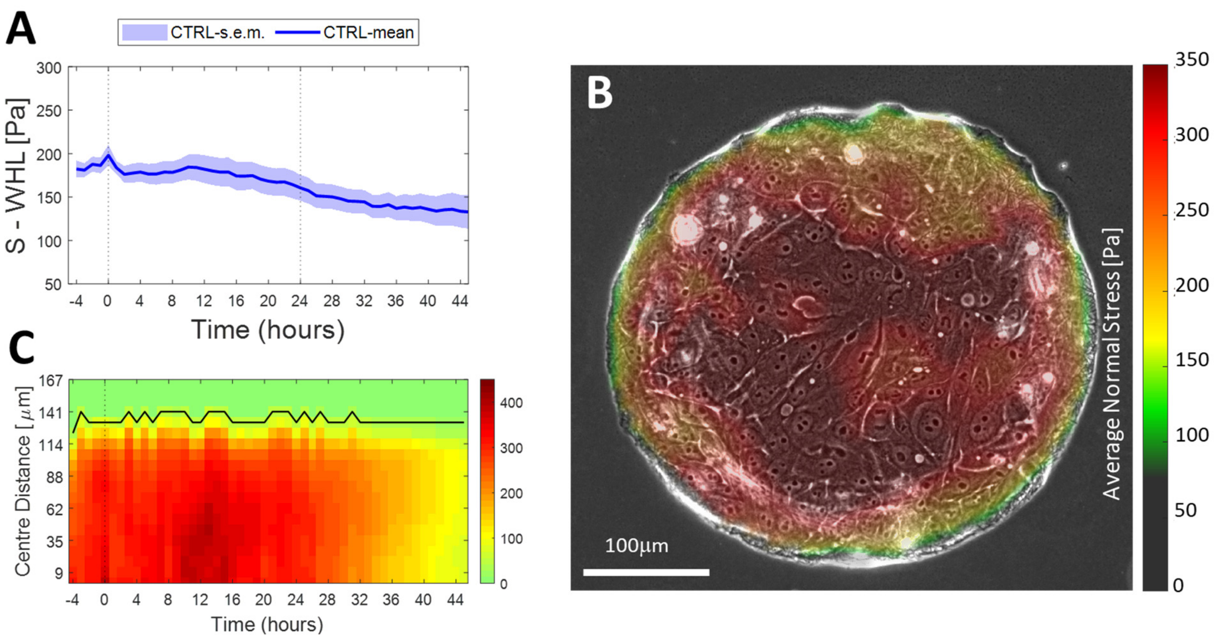


**Average normal stress within the non-transformed MCF10A monolayer.**

**A)** Time-evolution (Mean±S.E.M.) of average normal stress (Mean±S.E.M.) in the whole island's domain. Statistics over 15 non-transformed epithelia from at least 4 independent experiment repeats. **B)** Average normal stress within the epithelial monolayer (red is tension and grey is compression, according to the color bar). **C)** Kymograph of the average normal stress for a representative control MCF10A circular epithelium. Black line represents the average evolution of the edge of the island in time, the center of the island co-localizing with the bottom of the graph.

**Fig. S5.**

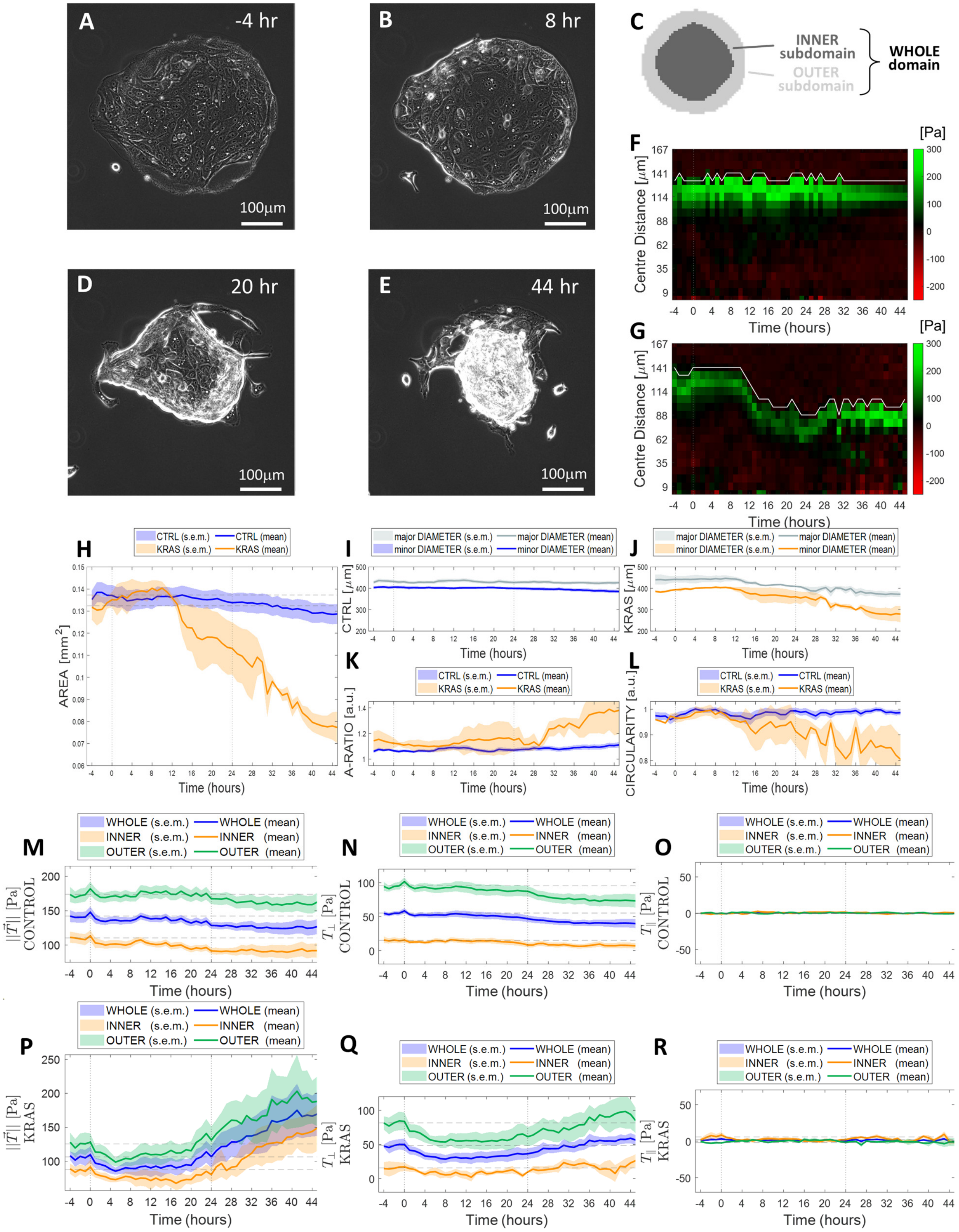
A
-4 hr
100µm
B
8 hr
100µm
C
INNER subdomain
OUTER subdomain
WHOLE domain
D
20 hr
100µm
E
44 hr
100µm
F
G
Centre Distance [µm]
Time (hours)
[Pa]
H
AREA [mm2]
CTRL (s.e.m.)
CTRL (mean)
KRAS (s.e.m.)
KRAS (mean)
I
J
major DIAMETER (s.e.m.)
major DIAMETER (mean)
minor DIAMETER (s.e.m.)
minor DIAMETER (mean)
CTRL [µm]
KRAS [µm]
K
A-RATIO [a.u.]
L
CIRCULARITY [a.u.]
M
N
O
P
Q
R
WHOLE (s.e.m.)
WHOLE (mean)
INNER (s.e.m.)
INNER (mean)
OUTER (s.e.m.)
OUTER (mean)
CONTROL
KRAS
[Pa]

**Morpho-mechanical evolution of KRAS-transformed MCF10A monolayers. A,B,D,E)** Phase contrast time-lapse of a *KRAS*-transformed MCF10A monolayer (imaging starts at t=-4 hours and *KRAS*-activation is induced at t=0 hours; scale bar 200 µm). **C)** Schematic representing the whole epithelial domain along with its outer and inner subdomains. **F-G)** Kymographs of the perpendicular component of the traction field $T_{\perp}$: **F)** for a representative non-transformed MCF10A epithelium; and, **G)** for a representative *KRAS* -transformed MCF10A epithelium. White lines represent the average evolution of the edge of the island in time, the center of the island co-localizing with the bottom of the graph. A negative component $T_{\perp}$ (red) means that the corresponding traction-force vector is in oriented towards the exterior of the epithelial domain's edge, whereas a positive component $T_{\perp}$ (green) is indicative of traction-force orientation toward the interior of the epithelial domain's edge. **H-L)** Time-evolution of the: **H**) surface area; **I-J**) major and minor diameters of the epithelial domain in the case of non-transformed (I) and *KRAS* -transformed (J) tissues; **K**) epithelial domain's aspect ratio; and, **L**) epithelial domain's circularity. **M,P)** Time evolution (Mean±S.E.M.) of the average Traction-field magnitude computed as $\|\vec{\boldsymbol{T}}\|$, the Euclidean norm (length) of the traction-force vectors of the field, for both: **M)** non-transformed epithelia; and, **P)** *KRAS* -transformed epithelia. **N-O,Q-R)** Traction-field components in the whole epithelial domain (blue), in the inner (orange) and outer (green) epithelial subdomains. Computed with their own sign by projecting traction-force vectors (at each time point and in each location of the epithelial domain) along the perpendicular and tangential directions respectively (Suppl. Fig. 3). Time evolution of the average traction force components: **N,Q)** perpendicular to the island's edge ($T_{\perp}$) for non-transformed (N) and *KRAS*-transformed epithelia (Q); **O,R)** tangential to the island's edge ($T_{\parallel}$) for non-transformed (O) and *KRAS* -transformed epithelia (R).

**Fig. S6.**

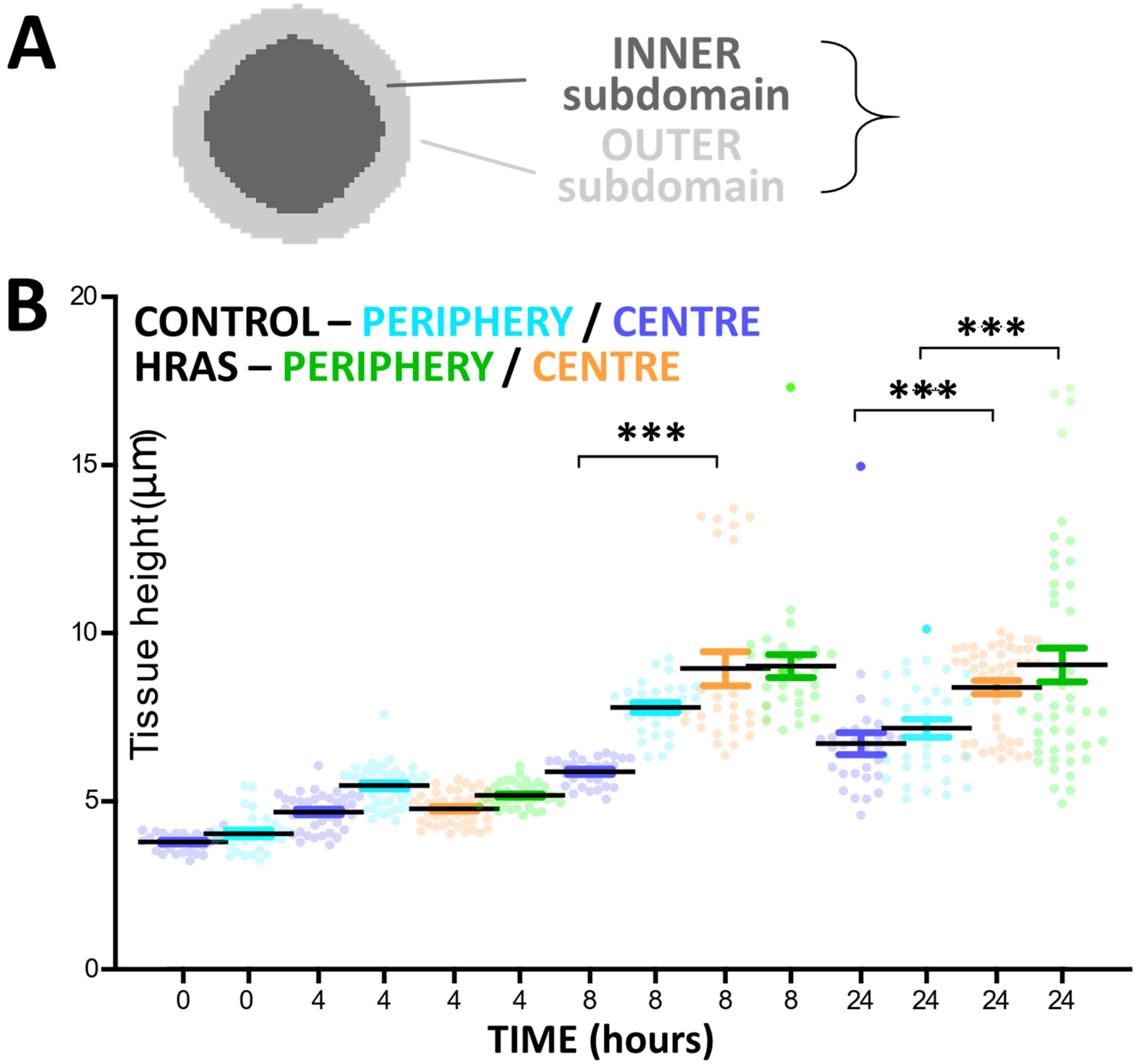


**Epithelial thickness in the inner and outer epithelial domains.**

**A)** Schematic representing the whole epithelial domain along with its outer and inner subdomains. **B)** MEAN±S.E.M. of measurements from at least 3 individual patterns. Kruskal-Wallis statistic test with Dunn's Multiple Comparison Test ***p<0.001.

**Fig. S7.**

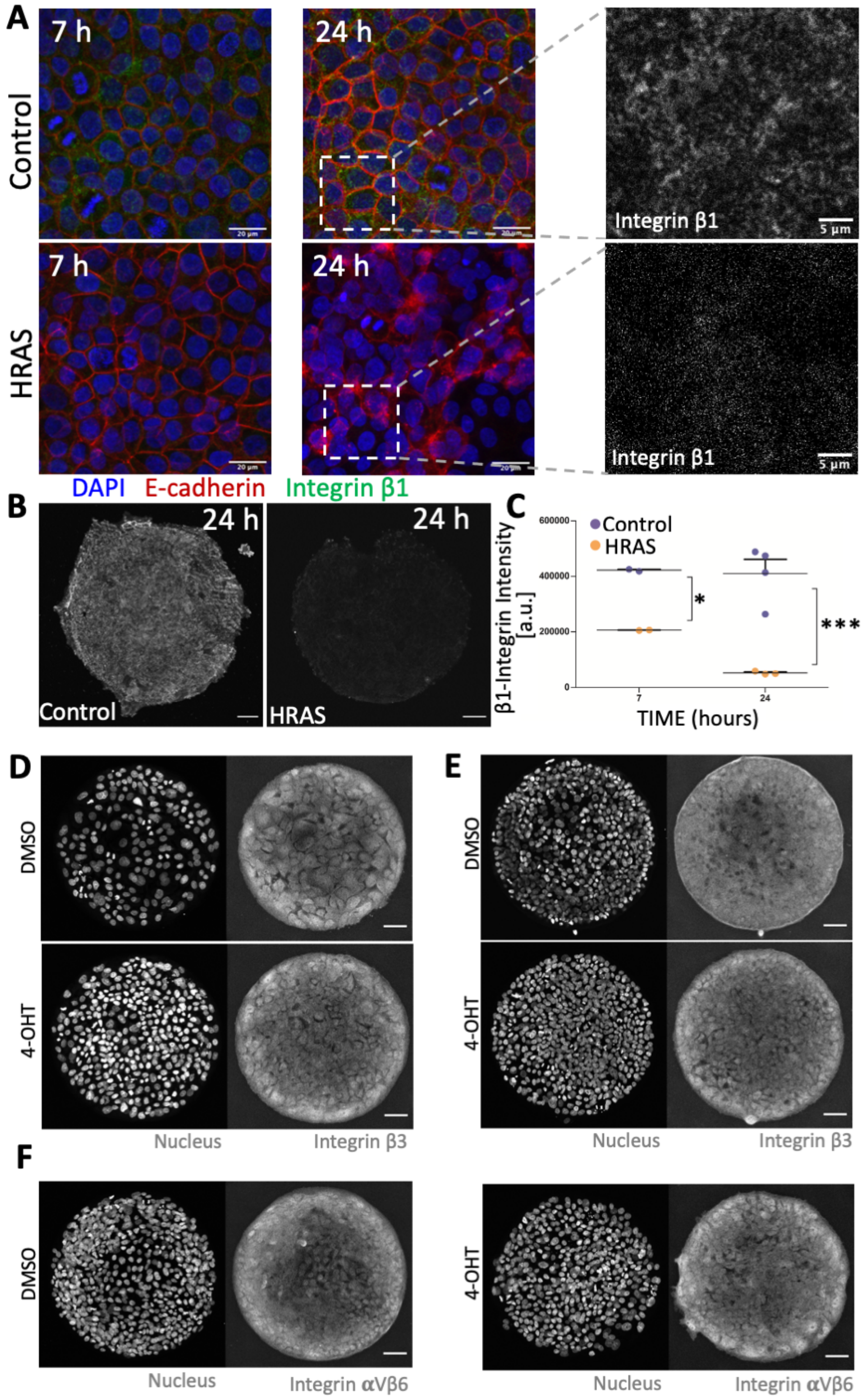
A
7 h
24 h
Control
HRAS
Integrin β1
5 µm
DAPI E-cadherin Integrin β1
B
24 h
Control
HRAS
C
Control
HRAS
β1-Integrin Intensity [a.u.]
600000
400000
200000
0
*
***
7
24
TIME (hours)
D
E
DMSO
4-OHT
Nucleus
Integrin β3
F
Integrin αVβ6

**HRAS-expression alters the expression of β1-integrin.**

(**A**) Confocal images of non-transformed and *HRAS*-transformed epithelia after 7 and 24 hours of oncogene induction stained for E-cadherin (red), DAPI (blue) and integrin β1 (green in panels at the center and shades of greys in panels on the right). Scale bar = 20 µm (**B**) Confocal images of the entire non-transformed monolayer and *HRAS*-transformed bilayer after 24 hours of oncogene induction stained for integrin β1 (shades of greys) – focal plane crosses the tissue basally and parallelly to the substrate matrix. Scale bar = 50 µm. (**C**) Intensity of global β1-integrin fluorescence in non-transformed and *HRAS*-transformed epithelia after 7 and 24 hours of oncogene induction. Mean± S.E.M.; 2-way ANOVA with Bonferroni post-test. *p<0.05, ***p<0.001. (**D-E**) Confocal images of the entire non-transformed monolayer and *HRAS*-transformed bilayer after 24 hours of oncogene induction stained for integrin β3 (MAB2023Z, Merck (**D**)), integrin β3 (anti-CD61 antibody, 16-0611-82, eBioscience, (**E**)), integrin αVβ6 (MAB2077Z, Merck, (**F**)), co-stained with nuclei (shades of greys) – focal plane crosses the tissue basally and parallelly to the substrate matrix. Scale bar = 50 µm.

**Table S1.**

| | *t* = -4h | *t* = 0h | *t* = 24h |
|---|---|---|---|
| **cell-matrix adhesion weakening factor $\alpha$** | 1.00 | 1.00 | 0.30 |
| **epithelial baseline contractile pre-strain $\varepsilon_0^c$** | -0.50 | -0.50 | -0.50 |
| **cell active contractile strain $\varepsilon^c$** | 0.00 | 0.00 | -0.45 |
| **total cell-cell contractile strain $\varepsilon = \varepsilon_0^c + \varepsilon^c$** | -0.50 | -0.50 | -0.95 |

In silico adhesion and contractility parameters.

**Movie S1.**

**Time-lapse of phase-contrast images of non-transformed MCF10A epithelial monolayer.** Micropatterned MCF10A ER:HRAS treated with DMSO (control) monitored over 50 hours (scale bar = 100 μm).

**Movie S2.**

**Time-lapse of phase-contrast images of HRAS-transformed MCF10A epithelial monolayer.** Micropatterned MCF10A ER:HRAS treated with 4-OHT (RAS-transformed) monitored over 50 hours (scale bar = 100 μm).

**Movie S3.**

**Time-lapse of traction maps of non-transformed MCF10A epithelial monolayer.** Color map represents the component normal to the center of the monolayer, imposed on phase contrast images (scale bar = 100 μm). Total duration: 50 hours.

**Movie S4.**

**Time-lapse of traction maps of HRAS-transformed MCF10A epithelial monolayer.** Color map represents the component normal to the center of the monolayer, imposed on phase contrast images (scale bar = 100 μm). Total duration: 50 hours.
